# The Effect of Shionone on Sepsis-Induced Acute Lung Injury by the ECM1/STAT5/NF-κB Pathway

**DOI:** 10.3389/fphar.2021.764247

**Published:** 2022-01-26

**Authors:** Yi Song, Qian Wu, Huojun Jiang, Aihao Hu, Lingqi Xu, Caiping Tan, Biao Zhang, Rongming Yu, Yizhen Qiu, Xin Wang, Wenzhong Yang

**Affiliations:** ^1^ Department of Critical Care Medicine, Suzhou Hospital of Integrated Traditional Chinese and Western Medicine, Suzhou, China; ^2^ Li Shicai School Inheritance Studio, Suzhou Hospital of Integrated Traditional Chinese and Western Medicine, Suzhou, China

**Keywords:** shionone, sepsis-induced acute lung injury, macrophage polarization, ECM1/STAT5/NF-κB pathway, *Aster tataricus*

## Abstract

**Purpose:** The purpose of the present study was to estimate the effect of shionone (SHI) on sepsis-induced acute lung injury (ALI).

**Methods:** The cecal ligation and puncture (CLP) surgery was performed to induce sepsis in mice. Pulmonary hematoxylin and eosin staining, the wet/dry ratio, myeloperoxidase (MPO) activity, and the survival rate were detected. The RAW264.7 cells were treated with SHI and stimulated with lipopolysaccharide (LPS). The cells were also overexpressed by extracellular mechanism protein 1 (ECM1) adenovirus. The relative levels of granulocyte–macrophage colony-stimulating factor, IL-6, IL-1β, TNF-α, IL-10, and TGF-β in the serum and supernatant were measured by ELISA. The protein expressions of ECM1, p-STAT5, signal transducer and activator of transcription 5 (STAT5), p-NF-κB, nuclear factor kappa-B (NF-κB), Arg1, CD206, CD16/32, and iNOS in the CLP-induced lung tissues and LPS-induced cells were detected by western blot. The cell counts of Ly6G, F4/80, CD16/32, and CD206 were evaluated by flow cytometry. The ECM1 expression was also observed by immunohistochemistry and immunofluorescence staining.

**Results:** As a result, the histopathological change, pulmonary edema, and the MPO activity were relieved by SHI. SHI treatment increased the percentage of neutrophil and macrophage in the bronchoalveolar lavage fluid. Besides, SHI administration inhibited pro-inflammatory cytokines and M1 phenotype indices, as well as augmented the anti-inflammatory cytokines and M2 phenotype indices. SHI also attenuated the ECM1/STAT5/NF-κB pathway both *in vivo* and *in vitro*. The overexpression of ECM1 confirmed that the regulated effect of SHI was due to ECM1 signaling.

**Conclusion:** In conclusion, the present study suggests that SHI ameliorated sepsis-induced ALI by screwing M1 phenotype to M2 phenotype macrophage via the ECM1/STAT5/NF-κB pathway.

## Introduction

Sepsis is a severe disorder featured by overwhelming systemic inflammatory reaction and causes various organ failures which are even lethal. Cecal ligation and puncture (CLP) is the commonly used method to induce polymicrobial sepsis containing gram-negative bacteria–related sepsis. The cecum ligated area and puncture number are responsible for the intensity of sepsis (Zhang et al., 2021a). The peripheral injection of bacterial endotoxin lipopolysaccharide (LPS) is also frequently applied to mimic sepsis in the murine model. LPS, the outer membrane component of gram-negative bacteria, is the famous endotoxin to induce overproduction of pro-inflammatory cytokines (Chen et al., 2020).

As one of the major complication of sepsis, acute lung injury (ALI) is the devastating disease characterized by respiratory failure, alveolar–capillary membrane barrier, pulmonary edema, and immune/inflammatory reaction. ALI and its severe form the acute respiratory distress syndrome are the important factors contributing to the death of sepsis patients. The neutrophils and macrophages are major cells located in the alveoli and are frequently found in the bronchoalveolar lavage fluid (BALF). The activation of neutrophil and macrophage is the pathological hallmark of the immune/inflammatory response in ALI (Fowler et al., 2019; Zhang et al., 2021a). Despite huge efforts being made, the etiology of sepsis-induced ALI is still not fully understood. Most clinical therapeutic methods are conducted to prevent the symptom of sepsis-induced ALI, whereas its specific drugs are warranted.

Upon the initiation of inflammation, the macrophage polarizes to classically activated phenotype (M1) and alternatively activated type (M2) in different stimuli conditions. M1 microphage is induced by LPS and releases the pro-inflammatory mediators including tumor necrosis factor-α (TNF-α), interleukin-1β (IL-1β), and IL-6. The biomarkers of the M1 macrophage include CD16/32 and iNOS. It is universally acknowledged that M1 phenotype aggravates the pathophysiology of inflammatory dysfunctions. The M2-type macrophage, secreting anti-inflammatory cytokine IL-10, is marked by CD206 and arginase (Arg1). In the normal situation, most tissue resident macrophages exert M2-like characteristics. The M2 macrophage is also induced by harmful stimuli and repairs the tissue damage. The promotion of the M2 macrophage and the suppression of the M1 macrophage has been reported to restrain the symptom of sepsis-induced ALI (Janicova et al., 2019; Hu et al., 2021). CLP and LPS initiate nuclear factor kappa-B (NF-κB) which governs M1 phenotype macrophage activation. Extracellular mechanism protein 1 (ECM1) is highly expressed in macrophages; ECM1 inhibits colony-stimulating factor 2 [granulocyte–macrophage colony-stimulating factor (GM-CSF)] and further augments the phosphorylation of signal transducer and activator of transcription 5 (STAT5), which consequently screws the macrophage to the M2 site (Zhang et al., 2020).

The Chinese herb *Aster tataricus* is distributed in eastern Asia and exhibits a protective effect on respiratory disease, such as pharyngitis, cough, and asthma. Shionone (SHI) is the triterpenoid ingredient extracted from the dried root and rhizome of *A. tataricus* (Tian et al., 2009; Yu et al., 2015). It represses the growth, migration, and invasion of breast cancer by influencing the phosphorylation of STAT3 (Xu et al., 2020). It has been illustrated that SHI shows anti-virus and anti-immune responses (Zhou et al., 2014). SHI has also been verified to exert anti-inflammatory activity by suppressing the expressions of pro-inflammatory cytokine, as well as the activation of NLRP3 inflammasome and nuclear factor kappa B (NF-κB) (Wang et al., 2021). It seems that SHI may be beneficial for pulmonary inflammatory disorders, including sepsis-induced ALI. However, the effect of SHI on ALI or sepsis remains elusive. The present research was carried out to evaluate the effect of SHI on CLP-induced sepsis and LPS-induced RAW264.7 cells. Its potential mechanism was also explored both *in vivo* and *in vitro*.

## Materials and Methods

### Reagents

SHI, with purity over 98%, was obtained from Shanghai Yuanye Bio-Technology Co., Ltd. Dexamethasone (DXM) was supplied from Xiansheng Drug Store (Nanjing, China). The drugs were dissolved by DMSO [with the concentration of DMSO less than 0.1% (w/v)]. LPS (*Escherichia coli* 055:B5) was produced by Sigma (St. Louis, United States). Mouse TNF-α, IL-1β, IL-6, IL-10, and TGF-β were purchased from Elabscience (Wuhan, China). The myeloperoxidase (MPO) commercial kit was provided by Jiancheng (Nanjing, China). The primary antibodies ECM1, Ly6G, F4/80, p-STAT5, STAT5, p-NF-κB, NF-κB, CD16/32, iNOS, CD206, Arg1, and GAPDH were produced by Cell Signaling Technology (CST) (Danvers, MA, United States).

### Animals

Male ICR mice (8 weeks old) were purchased from Qinglongshan Animal Farm (Nanjing, China). The animals were housed in standard laboratory at 24 ± 1°C with 40–60% and 12 h/12 day/night cycle. The mice were allowed to adapt to the environment 1 week before the experiment. All experiments were conducted according to the guide for the Care and Use of Laboratory Animals of NIH. Every effort was achieved to reduce the sacrifice and suffering of the mice.

### Cecal Ligation and Puncture–Induced Sepsis Procedure and Drug Treatment

The mice were randomly assigned to the sham, CLP, CLP + DXM (5 mg/kg), CLP + SHI (300 mg/kg), and CLP + SHI (600 mg/kg) groups. CLP surgery was employed to induce the murine sepsis model. After anesthesia, midline laparotomy was carried out on the mice, and the cecum was revealed. The feces were pushed to the cecum and fixed by a 5-0 propylene line. Any intestinal obstruction was avoided. The cecum was punctured 10 times to leak a small amount of faex. After that, the cecum was replaced in the abdominal cavity, and the wound was sutured. The sham mice suffered the same surgical process without puncturing. The mice were intragastrically treated with drugs 2 h before the surgery, and at 0, 2, and 12 h after the surgery. The sham and CLP mice were intragastrically treated with the vehicle at the same time. Their survival condition was recorded during the 48 h after the surgery. Then, the mice were sacrificed. After the sacrifice, the mice were lavaged with a tracheal cannula three times. A total volume of 1.3 ml of the BALF was obtained. The wet-to-dry weight (W/D) ratio was determined. The lung tissues were harvested and fixed in 4% paraformaldehyde and stored at −80°C for the pending test.

### Wet-to-Dry Weight Ratio

The lungs of the sacrificed mice were removed and weighted as wet weight (W). The lungs were then dried at 60°C for 48 h and weighted as dry weight (D). Consequently, the W/D ratio was measured to examine edema of the lungs.

### Myeloperoxidase Activity Assay

The lungs were homogenized and the protein concentration was examined by using the BCA commercial kit. The pulmonary MPO activity was assessed in accordance with the instrument. The absorbance change was determined at 460 nm using a full wavelength microplate reader.

### Hematoxylin and Eosin Staining

The lung tissues were fixed in 4% paraformaldehyde, imbedded with paraffin, and sliced. The sections were deparaffinized, rehydrated, and then stained by hematoxylin and eosin (H&E). The pulmonary histopathological alteration was observed under a light microscope.

### Cell Culture and Treatment

Murine RAW264.7 macrophage cells were supplied by American Type Culture Collection. The cells were cultured in Dulbecco's Modified Eagle Medium containing 10% heat-inactivated fetal bovine serum, 100 U/ml penicillin, and 100 μg/ml streptomycin at 37°C atmosphere with 5% CO_2_ and 95% air. The passage was carried out every 2–3 days. Only the cells in the logarithmic growth period were applied for the experiment in the present research.

A total of 5 × 10^4^ cells/ml were seeded onto six-well or 96-well plates. About 24 h later, the culture medium was renewed. The RAW264.7 cells were assigned into the control, LPS, LPS + SHI (0.5 μg/ml), LPS + SHI (1 μg/ml), and LPS + SHI (2 μg/ml) groups. The cells were treated with SHI (0.5, 1.0, and 2.0 μg/ml). About 2 h later, the cells were stimulated with LPS (5 μg/ml) for 24 h. The supernatant and cells were collected for further experiments.

The overexpressed adenovirus of ECM1 was provided by Heyuan Bio-Technology Co., Ltd. The RAW264.7 cells were divided to the control, SHI, overexpression (OE), and OE + SHI groups. A total of 5 × 10^2^ cells/ml were seeded onto six-well plates. About 24 h later, the culture medium was renewed. The ECM1 adenovirus was added for 24 h. Afterward, the cells were treated with SHI (2.0 μg/ml) for 2 h and then stimulated with LPS for 24 h. The cells were collected for pending experiments.

### MTT

A total of 5 × 10^4^ RAW264.7 cells/ml were seeded onto 96-well plates for 24 h. The cells were incubated with SHI (0.5, 1.0, and 2.0) for 2 h, and then with LPS (5 μg/ml) for another 24 h. The MTT solution (5 mg/ml) was added for 4 h. Thereafter, the culture medium was removed, and DMSO was added to dissolve formazan. The optical density value was detected at 570 nm using a full wavelength microplate reader.

### ELISA

The concentrations of GM-CSF, IL-1β, IL-6, TNF-α, IL-10, and TGF-β were measured by using commercial ELISA kits by following the manufacturer’s instruction (Elabscience, Wuhan, China).

### Flow Cytometry

The cells were collected and centrifuged at 6,000 *g* for 5 min at room temperature. To measure the cell surface markers of macrophage polarization, the cells were stained using anti-Ly6G-PE and anti-F4/80-FITC at 4°C for 30 min in the dark environment. The stained samples were detected using FACSCalibur (BD Bioscience, San Jose, United States) and analyzed using the FlowJo software (Tree Star Inc.).

### Western Blot

The lung tissues and RAW264.7 cells were lysed by RIPA. The protein concentration was determined by using the BCA Protein Assay Kit (Beyotime, Nanjing, China). The extract of protein was boiled at the equal amount. The sample was electrophoresed by 8–12% SDS-PAGE and electrotransferred onto the polyvinylidene diflouride membrane. The membrane was blocked at room temperature for 2 h by 3% bovine serum albumin (BSA) and then incubated with specific antibody at 4°C overnight. After washing with TBST, the blot was incubated with horseradish peroxidase–labeled secondary antibody at room temperature for 2 h on the shaker. Consequently, the immunoreactivity was visualized using enhanced chemiluminescence reagent.

### Immunohistochemical Staining

The lung tissues were immersed into 4% paraformaldehyde. The slides were dewaxed by xylene and exposed to gradient concentration ethanol. After antigen retrieval using citrate buffer at 95°C, the sections were quenched by endogenous peroxidases and blocked by 2% BSA for 30 min. The slides were incubated with primary antibody ECM1 followed by secondary antibody. The sections were stained with DAB and then with hematoxylin. An Olympus BX53 microscope (Olympus, Japan) was used for visualization. The relative ECM1 expression was determined by ImageJ software.

### Immunofluorescence Staining

The cells were stained with 4% paraformaldehyde and then blocked using 3% BSA supplemented with 0.1% Triton X-100. The samples were incubated with anti-Arg1 antibodies at 4°C overnight. After washing, the cell was incubated with Alexa Fluor® 488 secondary antibody (ab150077, Abcam, Cambridge, United Kingdom) in dark condition at room temperature for 1 h. The nuclei were stained with DAPI for 10 s. The samples were exposed to anti-fluorescence quenching sealing solution (Beyotime, Nanjing, China), and Arg1 expression was observed under the fluorescence microscope. Finally, the fluorescence intensity was calculated by using ImageJ and the representative image was observed.

### Statistical Analysis

Our data are depicted as mean ± SD. The results were analyzed by one-way ANOVA and followed up with Tukey’s multiple comparison test. *p* < 0.05 was considered as statistically significant.

## Results

### The Effect of Shionone on Sepsis-Induced Acute Lung Injury

**FIGURE 1:**
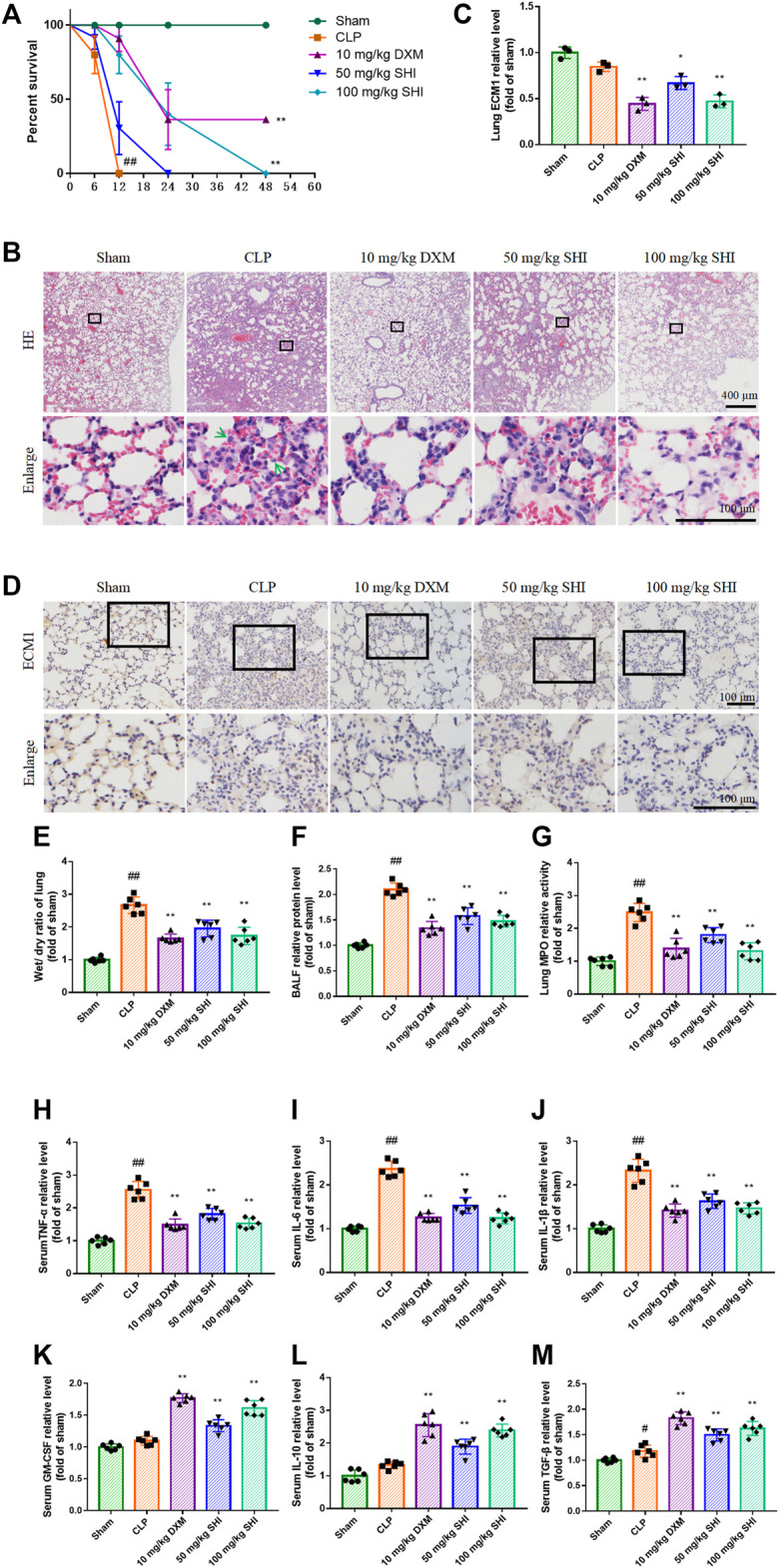
The effect of SHI on sepsis-induced ALI. The mice were intragastrically treated with drugs 2 h before the surgery, and at 0, 2, 12 h after the CLP surgery. **(A)** The survival percentage of sepsis mice. **(B)** The histopathological alteration by H&E staining. The green arrows indicate the infiltration of inflammatory cells. **(C,D)** ECM1 expression in the lung tissues by immunohistochemistry staining. **(E)** The wet/dry ratio of the lungs. **(F)** The BALF relative protein levels. **(G)** The lung MPO activity. The serum relative levels of **(H)** TNF-α, **(I)** IL-6, **(J)** IL-1β, **(K)** GM-CSF, **(L)** IL-10, and **(M)** TGF-β. The data are expressed as mean ± SD. Compared with sham: ^#^ *p* < 0.05, ^##^ *p* < 0.01. Compared with CLP: *p < 0.05, **p < 0.01.

The survival rate was monitored during the experiment. All mice in the sham group survived after 48 h of surgery. The animals in the CLP group all died within 12 h, and the animals in the SHI (50 mg/kg) group all died within 24 h. The survival percent in the SHI (100 mg/kg) and DXM groups was effectively reversed compared with that of the CLP group (*p* < 0.01) (Figure 1A). The histopathological alteration was observed by H&E staining. As revealed in Figure 1B, the CLP group presented inflammatory cell infiltration, pulmonary edema, and alveolar wall thickening compared with the sham group. The administrations of SHI (100 mg/kg) and DXM alleviated the pathological symptom compared with those in the CLP group, which seemed to be more obvious than in the SHI (50 mg/kg) group. The ECM1 expression in the CLP group was slightly inhibited compared with that of the sham group, by immunohistochemistry observation (Figures 1C,D). The treatment with SHI (100 mg/kg) and DXM further suppressed ECM1 expression (*p* < 0.01), which were more potent than in the SHI (50 mg/kg) group (*p* < 0.05). Besides, CLP surgery significantly increased the wet/dry ratio compared with that in the sham group (*p* < 0.01). The administration of SHI and DXM markedly reduced the pulmonary wet/dry ratio compared with the CLP group (*p* < 0.01) (Figure 1E). The CLP challenge also elevated the BALF relative protein expression level and lung MPO activity (*p* < 0.01). While SHI and DXM treatments were capable of reducing these indices (*p* < 0.01) (Figures 1F,G), the CLP stimulation dramatically increased serum levels of TNF-α, IL-6, and IL-1β (*p* < 0.01), which were evidently decreased in the SHI and DXM groups (*p* < 0.01) (Figures 1H–J). The SHI and DXM administration notably increased the serum relative levels of GM-CSF, IL-10, and TGF-β compared with those in the CLP group (*p* < 0.01). CLP stimulation elevated the expression of TGF-β when compared with the sham group (Figures 1K–M). Our data implied that SHI could attenuate ALI in CLP-induced sepsis mice, which was possibly related to ECM1-associated polarization alteration.

### The Effect of Shionone on the Ratio of Neutrophils and Macrophages in Bronchoalveolar Lavage Fluid

**FIGURE 2:**
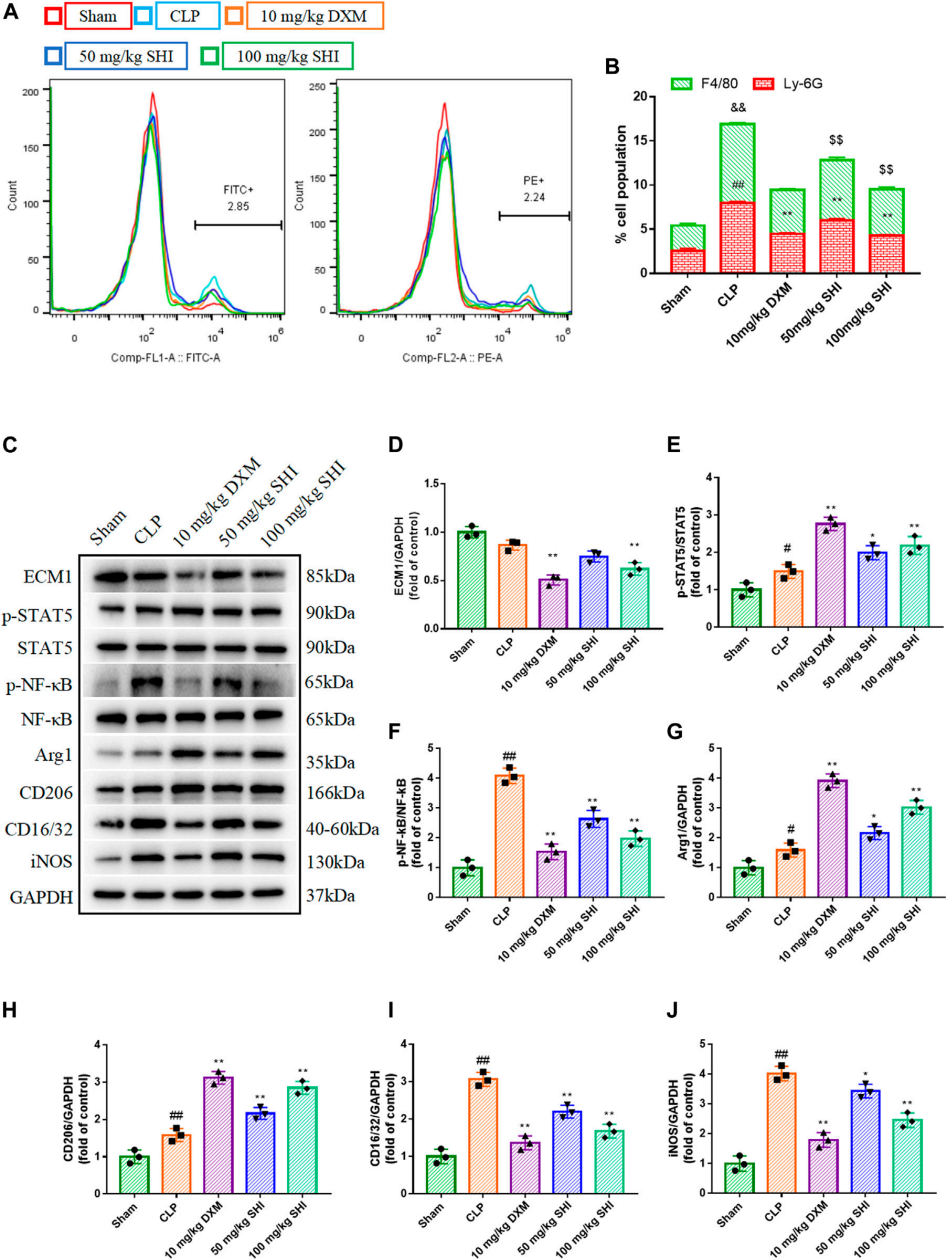
The effect of SHI on ECM1/STAT5/NF-κB signaling in CLP-induced mice. The mice were intragastrically treated with drugs 2 h before the surgery, and at 0, 2, 12 h after the CLP surgery, respectively. The neutrophils were stained by anti-Ly6G-PE, while the macrophages were stained by anti-F4/80-FITC. **(A,B)** The effect of SHI on the populations of neutrophils and macrophages in the BALF were detected by flow cytometry. **(C–J)** The protein expressions of ECM1, p-STAT5, STAT5, p-NF-κB, NF-κB, Arg1, CD206, CD16/32, and iNOS in lung tissues. The data are expressed as mean ± SD. Compared with sham: ^#^p < 0.05, ^##^ *p* < 0.01. Compared with CLP: *p < 0.05, **p < 0.01.

The ratio of the neutrophils and macrophages were detected using flow cytometry by anti-Ly6G PE and anti-F4/80 Alex Fluor488 (FITC), respectively. As presented in Figures 2A,B, the CLP exposure remarkably increased the neutrophils and macrophages ratio in the whole population of BALF cells (*p* < 0.01). Whereas, the SHI and DXM administrations all reduced the cell population percent of the neutrophils and macrophages in BALF (*p* < 0.01).

### The Effect of Shionone on ECM1/STAT5/NF-κB–Mediated Macrophage Polarization

To investigate the effect of SHI on the macrophage, the M1 polarization indicators CD16/32 and iNOS and the M2 polarization biomarkers CD206 and Arg1 were measured. CLP stimulation distinctly upregulated CD16/CD32 and iNOS expressions (*p* < 0.01), as well as CD206 and Arg1 (*p* < 0.01 and *p* < 0.05, respectively) expressions. The treatments with SHI (100 mg/kg) and DXM markedly downregulated CD16/CD32 and iNOS expressions (*p* < 0.01), while SHI (50 mg/kg) also inhibited the expressions of CD16/CD32 and iNOS (*p* < 0.01 and *p* < 0.05, respectively). Nevertheless, SHI (100 mg/kg) and DXM promoted the CD206 and Arg1 expressions when compared with the CLP group, and SHI (50 mg/kg) showed augmented effect on CD206 and Arg1 levels (*p* < 0.01 and *p* < 0.05, respectively) (Figures 2C,G–J). Furthermore, the ECM1/STAT5/NF-κB pathway was detected. CLP challenge upregulated the expressions of p-NF-κB and p-STAT5 (*p* < 0.05 and *p* < 0.01, respectively). The administration of SHI (100 mg/kg) and DXM inhibited the ECM1 expression (*p* < 0.01). SHI (100 mg/kg) and DXM elevated the phosphorylated levels of STAT5 compared with those of the CLP group (*p* < 0.01). SHI (50 mg/kg) also augmented the phosphorylation of STAT5 (*p* < 0.05). Both DXM and SHI administration reduced the protein levels of phosphorylated NF-κB (*p* < 0.01) (Figures 2C–F). Our results suggest that SHI could promote M2 macrophage polarization and inhibit M1 macrophage polarization possibly via the ECM1/STAT5/NF-κB pathway.

### The Effect of Shionone on Macrophage Polarization in Lipopolysaccharide-Induced RAW264.7 Cells

**FIGURE 3:**
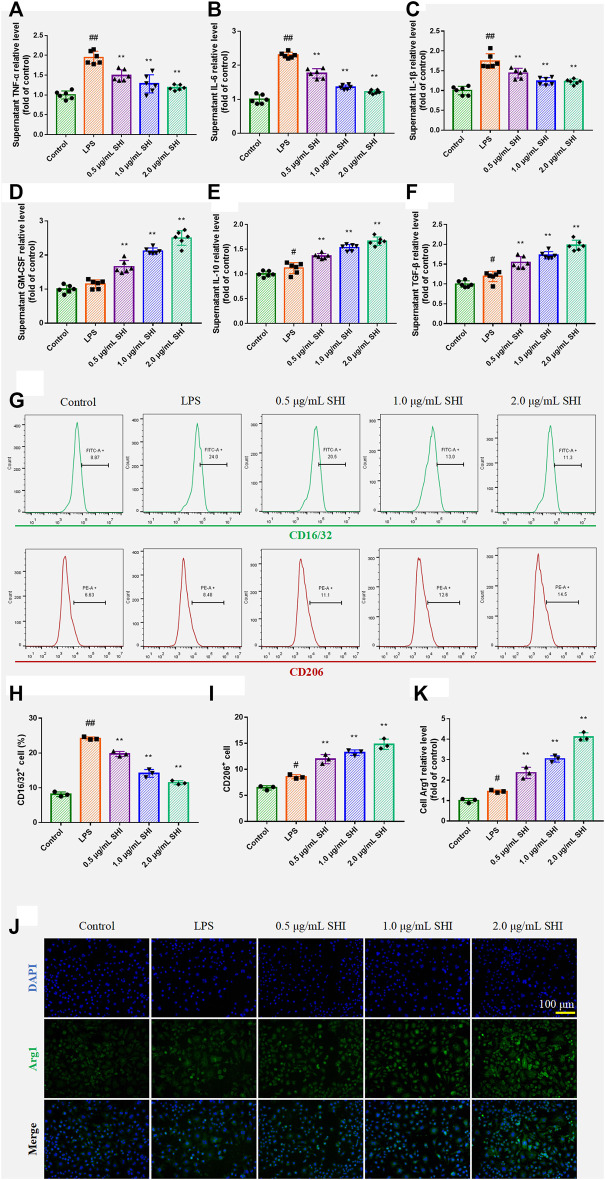
The effect of SHI on macrophage polarization in LPS-induced RAW264.7 cells. The cells were treated with SHI (0.5, 1.0, and 2.0 μg/ml). About 2 h later, the cells were stimulated with LPS (5 μg/ml) for 24 h. The relative levels of **(A)** TNF-α, **(B)** IL-6, **(C)** IL-1β, **(D)** GM-CSF, **(E)** IL-10, and **(F)** TGF-β in the supernatant of LPS-induced RAW264.7 cells. **(G–I)** The cell count of CD16/32 and CD206. **(J,K)** The ECM1 expression by immunofluorescence. The data are expressed as mean ± SD. Compared with control: ^#^ *p* < 0.05, ^##^ *p* < 0.01. Compared with LPS: *p < 0.05, **p < 0.01.

The SHI treatment increased the levels of GM-CSF (*p* < 0.01), which indicated that SHI could promote macrophage function (Figure 3A). The incubation with SHI apparently reduced the supernatant levels of TNF-α, IL-1β, and IL-6 (*p* < 0.01) (Figures 3B–D). On the other hand, SHI (1 and 2 μg/ml) treatment elevated the levels of IL-10 and TGF-β (*p* < 0.01), which exhibited stronger efficacy than SHI (0.5 μg/ml) treatment (*p* < 0.05) (Figures 3E,F). The CD16/32 and CD206 expressions were assessed by flow cytometry. As presented in Figures 3H–K, the LPS exposure obviously promoted the expressions of CD16/CD32 and CD206 (*p* < 0.05 and *p* < 0.01, respectively) compared with the control group. The incubation with SHI (0.5, 1, 2 μg/ml) downregulated CD16/CD32 expression and upregulated CD206 expression (*p* < 0.01) (Figures 3G–I). Arg1 expression was observed under the fluorescence microscope. SHI (0.5, 1, and 2 μg/ml) treatment promoted Arg1 expression (*p* < 0.01) (Figures 3J,K). The results suggest that SHI could inhibit M1 polarization biomarker expressions and promote M2 polarization biomarker expressions in LPS-induced macrophages.

### The Effect of Shionone on ECM1/STAT5/NF-κB Signaling in Lipopolysaccharide-Induced RAW264.7 Cells

**FIGURE 4:**
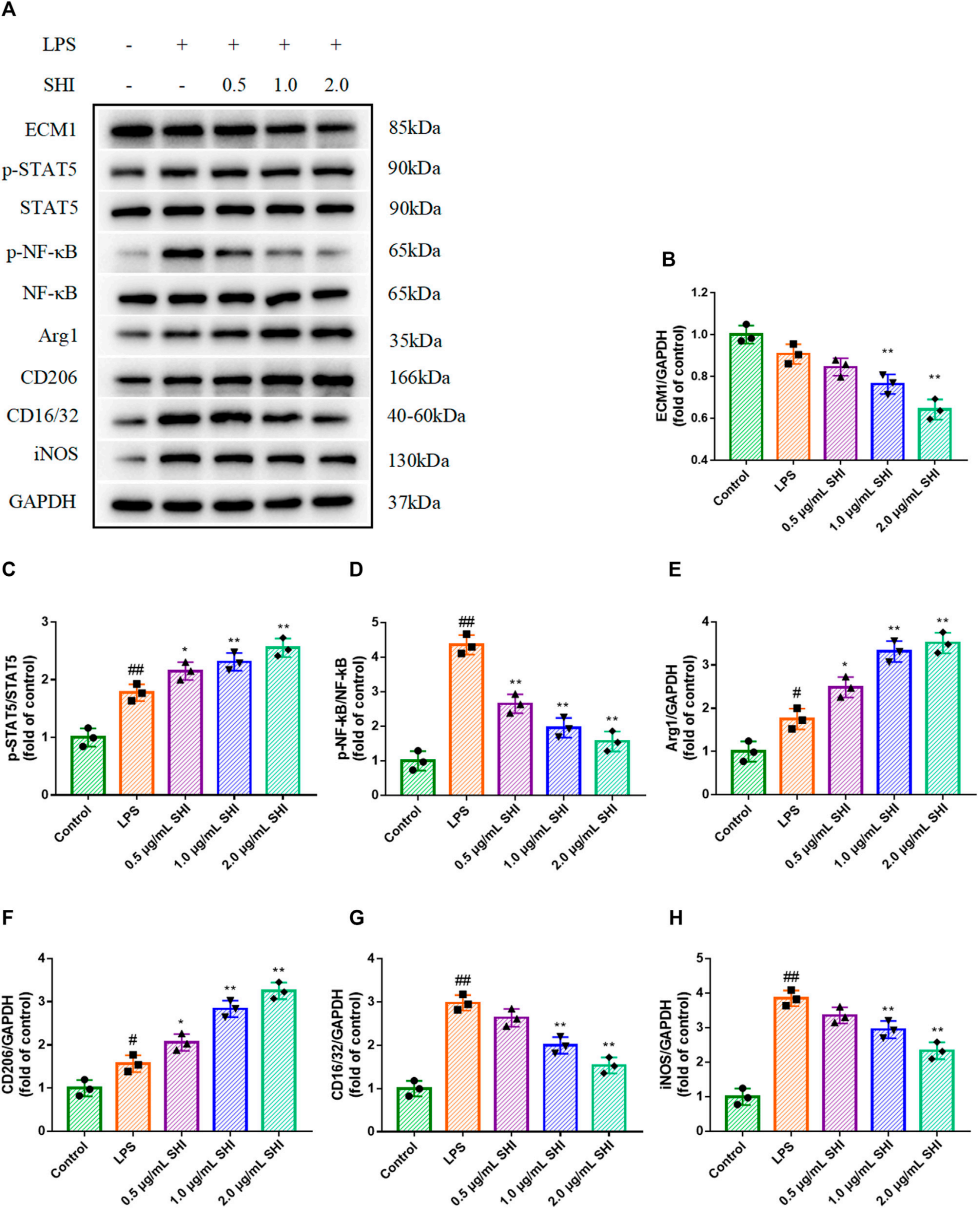
The effect of SHI on ECM1/STAT5/NF-κB signaling in LPS-induced RAW264.7 cells. The cells were treated with SHI (0.5, 1.0, and 2.0 μg/ml). About 2 h later, the cells were stimulated with LPS (5 μg/ml) for 24 h. **(A–H)** The protein expressions of ECM1, p-STAT5, STAT5, p-NF-κB, NF-κB, Arg1, CD206, CD16/32, and iNOS of LPS-induced RAW264.7 cells. The data are expressed as mean ± SD. Compared with control: ^#^ *p* < 0.05, ^##^ *p* < 0.01. Compared with LPS: *p < 0.05, **p < 0.01.

LPS challenge conspicuously increased Arg1 and CD206 expressions, as well as iNOS and CD16/32 expressions (*p* < 0.01). SHI (1 and 2 μg/ml) treatment enhanced Arg1 and CD206 expressions compared with those of the LPS group, which were stronger than SHI (0.5 μg/ml group) (*p* < 0.05) (Figures 4A,E,F). Additionally, the incubation with SHI (1 and 2 μg/ml) effectively blocked the iNOS and CD16/CD32 expressions (*p* < 0.01) (Figures 4A,G,H). LPS induction contributed to the phosphorylated STAT5 and NF-κB (*p* < 0.01). The treatment with SHI (1 and 2 μg/ml) further strengthened the STAT5 phosphorylation (*p* < 0.01), which was more efficient compared with that of the SHI (0.5 μg/ml) group (*p* < 0.05). The incubation with SHI (0.5, 1, and 2 μg/ml) prominently decreased the protein levels of p-NF-κB (*p* < 0.01) (Figures 4A–D). The results displayed that the modified effect of SHI on macrophage polarization might be attributed to the ECM1/STAT5/NF-κB pathway.

### The Role of Extracellular Mechanism Protein 1 in Shionone-Mediated Macrophage Polarization

**FIGURE 5:**
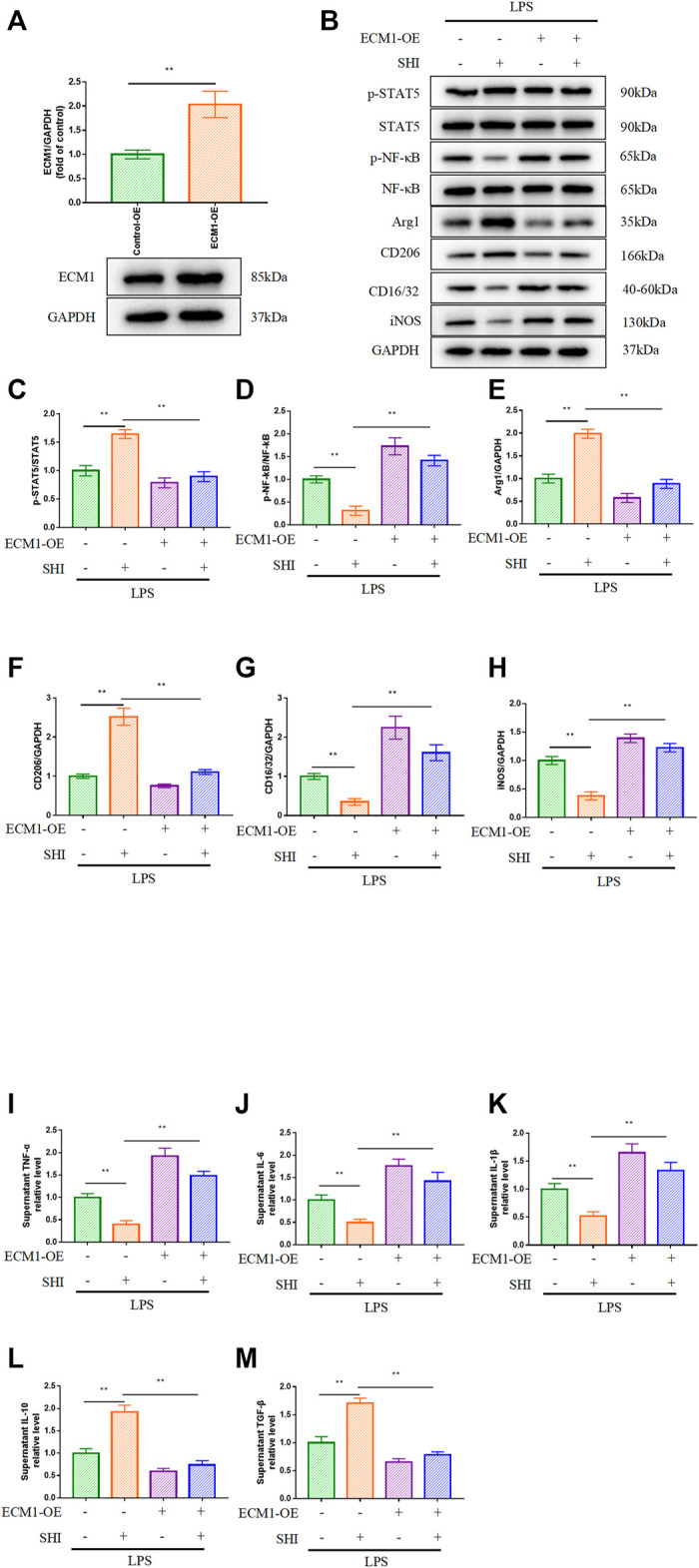
The role of ECM1 in SHI-mediated macrophage polarization. The cells were exposed to ECM1 adenovirus added for 24 h. Afterward, the cells were treated with SHI (2.0 μg/ml) for 2 h and then stimulated with LPS for 24 h. **(A)** The transfection efficiency of ECM1 overexpression. **(B–H)** The protein expression of ECM1, p-STAT5, STAT5, p-NF-κB, NF-κB, Arg1, CD206, CD16/32, and iNOS. **(I–M)** The supernatant levels of TNF-α, IL-6, IL-1β, IL-10, and TGF-β in LPS-induced RAW264.7 cells. The data are expressed as mean ± SD. Compared with the other group: *p < 0.05, **p < 0.01.

To further investigate the role of the ECM1/STAT5/NF-κB cascade in SHI-mediated macrophage polarization, we overexpressed ECM1, and the overexpression efficacy was confirmed in Figure 5A. As shown in Figures 4D–F, the overexpression of ECM1 pronouncedly inhibited p-STAT5, Arg1, and CD206 expressions and enhanced p-NF-κB, iNOS, and CD16/32 expressions (*p* < 0.01). It was noteworthy that the SHI-governed upregulations on p-STAT5, Arg1, and CD206 were hampered by ECM1 overexpression (*p* < 0.01) (Figures 5B,C,E,F). On the contrary, the SHI-mediated inhibitory effects on p-NF-κB, iNOS, and CD16/32 expressions were abrogated by ECM1 overexpression (*p* < 0.01) (Figures 5B,D,G,H). Additionally, the supernatant levels of TNF-α, IL-6, and IL-1β in the LPS + SHI group were distinctly decreased compared with those in the LPS group (*p* < 0.01), while the co-treatment with ECM1 adenovirus and SHI elevated the supernatant levels of these pro-inflammatory cytokines compared with the LPS + SHI group (*p* < 0.01) (Figures 5I–K). The supernatant contents of IL-10 and TGF-β were upregulated by the LPS challenge (*p* < 0.01). By contrast, the supernatant contents of the anti-inflammatory cytokines in the LPS + ECM1 overexpression + SHI group were obviously downregulated compared with those in the LPS + SHI group (*p* < 0.01) (Figures 5L,M). The experimental results displayed that SHI-shifted M1 to M2 macrophage polarization was mediated via the ECM1/STAT5/NF-κB signaling in LPS-induced RAW264.7 cells.

## Discussion

The high concentrations of pro-inflammatory cytokines in the serum result in multi-organ damage or even “systemic inflammatory response syndrome” in sepsis patients and animals. Sepsis also led to the accumulation of neutrophils and phagocytic cells. The neutrophil influx from the vessel to lung tissue and induce pulmonary inflammation in sepsis. Excessive neutrophils even elaborate sepsis-caused tissue damage. The transmigration of the neutrophils is reflected by the MPO activity (Park et al., 2019). Bronchoalveolar lavage is commonly employed to estimate the respiratory disorder status. Our experimental results show that the CLP procedure conduced to pulmonary histopathological alteration, accumulation of neutrophils and macrophages in the BALF, enhance MPO activity, and increase survival percentage, which indicated that the sepsis-induced ALI model was successfully established. Whereas these changes were effectively reversed by SHI administration.

Macrophages are critical phagocytic cells in the immune system and are distributed in multiple tissues including the lungs. The macrophage handles danger stimuli, maintains homeostasis, modulates inflammatory reaction, and accelerates wound healing. The macrophage polarization is responsible for the occurrence and development of immune disorder. CD16/32 and CD206 are the surface markers of M1 type and M2 type macrophages, respectively. Arg1 is promoted in the alternative activated macrophage. Former literatures identified the upregulation of Arg1 in the submucosal inflammatory cell of asthma patients. IL-10 is not only regarded as the specific M2 macrophage marker but also the indicative biomarker for deactivated macrophage. The elevation of M2 macrophage increased the anti-inflammatory reaction and the survival rate in septic mice (Carestia et al., 2019). The shift from M1 phenotype macrophage to M2 phenotype macrophage showed anti-inflammatory condition in the BALF and serum of LPS-induced mice (Lin et al., 2020). The inflammatory disease including sepsis and ALI locked the macrophages in the M1 state. The regulation of macrophage polarization was the therapeutic strategy for sepsis-induced ALI (Liu et al., 2020).

NF-κB, a critical cytoplasmic transcription factor driving immune and inflammatory process, is stimulated by TNF-α, LPS, and other tissue inflammatory injury (Chen et al., 2021). NF-κB controls the macrophage polarization by governing the transcription of various inflammatory factors in sepsis and ALI (Zhang et al., 2021b). Signal transducer and activator of transcription (STAT) family is the pivotal signaling implicated with the proliferation, differentiation, and immune modulation in mammals (Rani and Murphy, 2016). The activation of NF-κB requires the cooperation of multiple events including STAT transcription factors. STAT5, a member of the STAT family, interacts with the cofactors or transcription factors including NF-κB (Gerloff et al., 2015). LPS-caused STAT5 activation is mainly governed by GM-CSF (Fejer et al., 2013). In response to LPS, STAT5 also mediates the nucleus translocation and inflammatory factor expression. Upon GM-CSF activation, the nucleus STAT5 accelerates the transcription of M2 macrophage–associated genes including Arg1 (Kamigaki et al., 2016). The phosphorylation of STAT5 promotes M2 polarization rather than M1 polarization (Kuroda et al., 2009). Our results demonstrate that STAT5 phosphorylation was accompanied by M2 phenotype polarization, while NF-κB phosphorylation was associated with M1 phenotype polarization both in CLP-induced mice and LPS-induced RAW264.7 cells. Herein, NF-κB and STAT5 might be the crucial proteins for SHI-mediated M1 and M2 polarization, respectively.

ECM1 is highly expressed in macrophages and the tissues infiltrated with inflammatory cells. The specific downregulation of ECM1 in macrophages caused the promotion of Arg1 and prevented the screwing to the M1 phenotype. As the extracellular matrix factor, ECM1 controls the expression of the inflammatory lymphocytes in the respiratory system (Rostila et al., 2020). The recombinant ECM1 rapidly guided the Erk1/2 and Akt activation (Hardy et al., 2019). The ECM1 deficiency prevented the pathological progression in inflammatory disease by upregulating Arg1 expression and presenting M2 macrophage phenotype characteristic (Yasen et al., 2021). Thus, we assume that ECM1 might serve as the essential modulator of macrophage polarization. Excessive ECM1 expression was found in lung adenocarcinoma patients (Zhang et al., 2015). ECM1 expression was also upregulated in hypoxia-stimulated respiratory dysfunction rats (Ramirez et al., 2012). Therefore, it was hypothesized that ECM1 was closely related to pulmonary disease. As illustrated above, ECM1 was the key modulator of STAT5 and M2 macrophage polarization. Our analytical results depict that the treatment with SHI inhibited NF-κB phosphorylation and M1 subset hallmarks both *in vivo* and *in vitro*. Furthermore, the overexpression of ECM1 hampered the SHI treatment–mediated upregulation of M2 phenotype biomarkers and downregulation of M1 phenotype biomarkers, indicating that ECM1 was involved in the regulatory effect of SHI on macrophage polarization.

In conclusion, the present research demonstrated that ECM1 attenuated sepsis-induced ALI by screwing M1 phenotype to M2 phenotype polarization in CLP-induced mice and LPS-induced macrophage RAW264.7 cells, which was mediated by the ECM1/STAT5/NF-κB pathway. It was beneficial to perform the experiment with transgenic mice and alveolar macrophages. Besides, the activation and chemotaxis mechanism of macrophages could be explored in further investigation.

## Data Availability

The original contributions presented in the study are included in the article/supplementary material; further inquiries can be directed to the corresponding authors.
